# Comprehensive Analysis of ATP6V1s Family Members in Renal Clear Cell Carcinoma With Prognostic Values

**DOI:** 10.3389/fonc.2020.567970

**Published:** 2020-10-30

**Authors:** Xiaojuan Li, Hao Li, Caihong Yang, Liu Liu, Sisi Deng, Mi Li

**Affiliations:** ^1^ Department of Nephrology, Tongji Hospital, Tongji Medical College, Huazhong University of Science and Technology, Wuhan, China; ^2^ Department of Orthopedics, Tongji Hospital, Tongji Medical College, Huazhong University of Science and Technology, Wuhan, China; ^3^ Cancer Center, Union Hospital, Tongji Medical College, Huazhong University of Science and Technology, Wuhan, China

**Keywords:** renal clear cell carcinoma, ATP6V1s, public databases, prognostic value, comprehensive analysis

## Abstract

ATP6V1s participate in the biological process of transporting hydrogen ions and are associated with various cancers in expression and clinicopathological features, while its role in kidney renal clear cell carcinoma is unknown. We aimed to demonstrate the relationship between ATP6V1s and kidney renal clear cell carcinoma. This study investigated the expression and roles of ATP6V1s in KIRC using Oncomine, The Cancer Genome Atlas, UALCAN, Human Protein Atlas, Clinical Proteomics Tumor Analysis Consortium, GeneMANIA, Tumor IMmune Estimation Resource, GEPIA databases. Low mRNA and protein expression of ATP6V1s members were found to be significantly associated with clinical cancer stages, nodal metastasis status, and patient’s gender in KIRC patients. Besides, lower mRNA expression of ATP6V1A, ATP6V1B2, ATP6V1C1, ATP6V1C2, ATP6V1D, ATP6V1E1, ATP6V1E2, ATP6V1F, ATP6V1G1, and ATP6V1H have shorter OS. Taken together, these results indicated that ATP6V1s family members could be a potential target in the development of anti-KIRC therapeutics and an efficient marker of the prognostic value of KIRC.

## Introduction

Renal cell carcinoma (RCC) is one of the common urinary system tumors, which accounts for about 2–3% of adult malignant tumors, and its incidence is increasing year by year (1). The most common pathological type is kidney renal clear cell carcinoma (KIRC), which accounts for about 70–80% of RCC (2, 3). Compared with renal papillary cell carcinoma and renal chromophobe cell carcinoma, KIRC shows a poorer prognosis and is more prone to metastasis (4). When KIRC appears to metastasize, its outcome is less favorable (5). The curative treatment of early KIRC is partial or radical nephrectomy. However, about 30% of patients have a recurrence after surgery (6, 7). Advanced KIRC can be treated with molecular targeted therapy and immunotherapy, but the long-term efficacy is still unsatisfactory (8). Due to the insidious onset and without obvious symptoms, 30% of patients had invaded adjacent tissues or metastasized at initial diagnosis. Therefore, screening effective biomarkers for the diagnosis, treatment and prognostic evaluation of KIRC is of great clinical significance.

Although various biomarkers have been considered to be related to KIRC, such as bone morphogenetic protein 8A (9) and Cripto-1 (10), their reliability remains controversial. Vacuolar adenosine triphosphatase (V-ATPase) is widely distributed in eukaryotic cells and transports H^+^ by hydrolyzing ATP. Studies have shown that V-ATPase affects tumor invasion and invasion. V-ATPase is divided into two parts, including located in cytoplasmic part V1 and transmembrane part V0. V-ATPase V1 is also called ATP6V1 (11), which is composed of subunits A–H (12). The main function of ATP6V1 is to hydrolyze ATP to provide energy for transporting H^+^. Numerous studies have shown that ATP6V1 plays an important role in diseases such as tumors, kidney diseases, abnormal bone development, and diabetes (11, 13, 14). However, few studies about the relationship between ATP6V1 and KIRC has been reported so far.

In this study, we addressed this problem by identifying the transcriptional and protein expression patterns of ATP6V1s family members *via* The Cancer Genome Atlas (TCGA), Oncomine, Clinical Proteomics Tumor Analysis Consortium (CPTAC), and Human Protein Atlas (HPA) databases. Then we continued to predict Gene Ontology functions and biological pathways of ATP6V1s together with their 20 related genes. Furthermore, we analyzed clinical features and prognostic values of ATP6V1s family members in KIRC. The current study shows the potential biological functionality and prognostic value of ATP6V1s, which will be beneficial to the diagnosis and treatment of kidney renal clear cell carcinoma.

## Materials and Methods

### Differentially Expressed ATP6V1s at the Transcriptional Level

Oncomine 4.5 (www.ocomine.org) is an integrated online oncogene microarray database and data-mining platform, which provides peer-reviewed, robust analysis methods, and a powerful set of analysis functions to compute gene expression signatures (15). In our study, the mRNA expressions of 8 different ATP6V1s family members in KIRC tissues with their corresponding adjacent normal control samples were analyzed by the Oncomine database. The data in our study were compared by the t-test and cut- off *p*-value and fold change were as following: *p*-value < 0.0001, fold change = 2, gene rank = 10%.

TCGA database (http://cancergenome.nih.gov/) is a comprehension and the coordinated project contains gene expression database and corresponding clinical information data (16). The gene expression of ATP6Vs in KIRC and corresponding clinical information data were downloaded from the TCGA database. UALCAN (http://ualcan.path.uab.edu) is a comprehensive and interactive web resource based on RNA-seq of 31 cancer types from the TCGA database (17). To determine the reliability of the differential expression data, the UALCAN database was selected for further verification. In this study, the mRNA expressions of different ATP6V1s family members of KIRC tissues and normal tissues were analyzed in the TCGA-KIRC dataset. *P* < 0.001 was considered statically significant.

### Differentially Expressed ATP6V1s at Protein Level

In addition to the TCGA and UALCAN database providing mRNA expression analysis of ATP6V1s family members, the protein expression analysis of ATP6V1s family members was provided using the data from the CPTAC Confirmatory/Discovery dataset for KIRC (18). The CPTAC is used for proteomics research of various tumors. In this work, the protein expressions of different ATP6V1s family members between KIRC tissues with normal tissues were analyzed following the CPTAC reproducible workflow protocol. *P* < 0.001 was considered statically significant.

HPA (http://www.proteinatlas.org) is a platform that contains representative immunohistochemistry-based protein expression data for near 20 highly common kinds of cancers (19). In this study, immunohistochemistry images of protein expression of different ATP6V1s family members between normal and KIRC samples were directly visualized by HPA.

### Construction of Related Genes Network

GeneMANIA 3.6.0 (http://www.genemania.org) is a website for generating hypotheses about gene function using available genomics and proteomics data (20). In our study, the ATP6V1s family members were submitted to the GeneMANIA to illustrate the functional association network among ATP6V1s and their related genes. The advanced statistical options were that max resultant attributes were 10, max resultant genes were 20, and the weighing method used was automatically selected.

### GO Enrichment Analysis and Kyoto Encyclopedia of Genes and Genomes (KEGG) Pathway Enrichment Analysis

DAVID (https://david.ncifcrf.gov/), is a functional enrichment analysis web tool with continuously updated and effectively reduce data redundancy (21). GO functions and pathways of ATP6V1s and their 20 related genes were enriched by WebGestalt. The Method of Interest is selected in Over-Representation Analysis (ORA). The GO functional enrichment was performed in the biological process no Redundant (BP), cellular component no Redundant (CC), molecular function no Redundant (MF). And the pathway analysis was performed in the KEGG pathway.

### Immune Infiltration Analysis of ATP6V1s

TIMER (https://cistrome.shinyapps.io/timer/) is a systematic database using the microarray expression values for calculating a comprehensive analysis of immune infiltrates through different cancer types (22). The immune infiltration estimation of ATP6V1s was performed in KIRC by TIMER. The scatterplots of ATP6V1s was generated to show the purity-corrected partial Spearman’s rho value and statistical significance. The positive purity value expected genes are highly expressed in the tumor cells, and the opposite is expected for genes highly expressed in the microenvironment.

### Clinicopathological Analysis of ATP6V1s in KIRC

Furthermore, UALCAN was used to analyze the association between the mRNA or protein expressions of ATP6V1s in KIRC tissues with their clinicopathologic parameters such as individual cancer stages, nodal metastasis status, and patient’s gender. The results could be got directly by selecting the clinicopathological grouping options integrated into the UALCAN database. In particular, only the tumor group could be divided into different clinicopathological groups. The statically significant *p* is less than 0.001.

### Survival Analysis

In this study, the prognostic value of mRNA expression of distinct ATP6V1s in KIRC was analyzed by GEPIA (http://gepia.cancer-pku.cn/index.html) (23), which contains 9,736 tumors and 8,587 normal samples from the TCGA and the GTEx. Based on the median values of mRNA expression, patients with KIRC were divided into high and low expression groups. *p* < 0.05 was considered statically significant.

### Statistical Analysis

All statistical analysis analyses and plots were produced using R (v.3.5.1). T-test was used to analyze the expression of ATP6V1s. One-way ANOVA test, Wilcoxon signed-rank test, and logistic regression were used to evaluate relationships between clinical-pathologic features and the expression of ATP6V1s. Cox regression analyses and the Kaplan-Meier method were used to evaluate prognostic factors.

## Results

### Low mRNA Expression of Different ATP6V1s Family Members in Patients With KIRC

**Figure 1:**
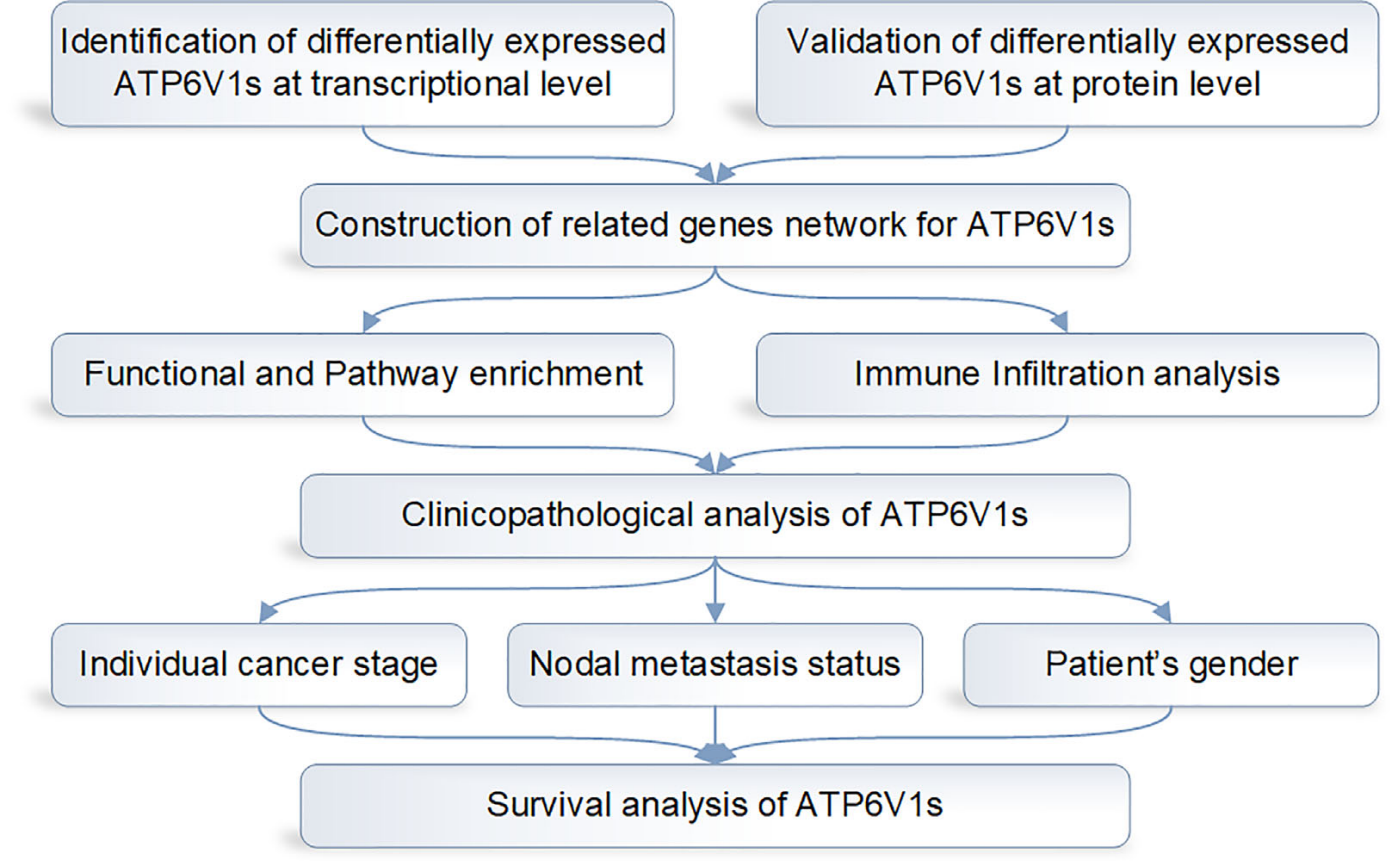
Design flow chart of the whole analysis process of this study.

The design flow chart of the whole analysis process of this study is shown in **Figure 1**.

**Figure 2:**
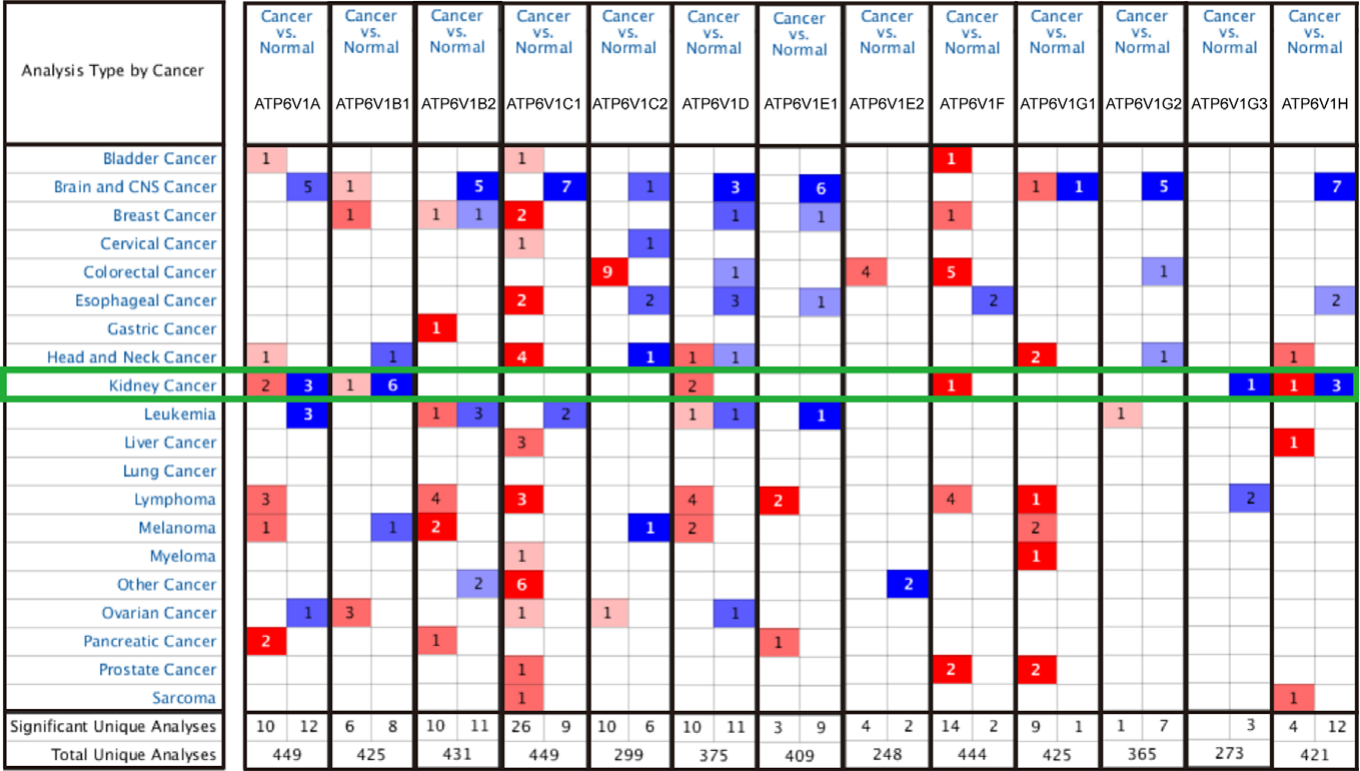
Transcriptional expressions of different ATP6V1s family members in 20 types of cancers (Oncomine database). Transcriptional expressions of different ATP6V1s family members of human cancers. The data were compared by the t-test and cut-off *p*-value and fold change were as following: *p*-value < 0.0001, fold change = 2, gene rank = 10%. Red indicates over-expression, blue indicates down-expression.

In order to research the mRNA expression of different ATP6V1s family members in RCC patients, data of 20 types of cancers were analyzed and compared to normal tissues by the Oncomine database. As shown in **Figure 2** and **Table 1**, mRNA expressions of ATP6V1A, ATP6V1B1, ATP6V1D, ATP6V1F, ATP6V1G3, and ATP6V1H were significantly higher in RCC tissues. In the Beroukhim KIRC dataset, the mRNA expression of ATP6V1A, ATP6V1B1, and ATP6V1H was lower in RCC tissues compared with normal tissues with fold changes of 2.403, 13.706, and 2.276 (*p*=4.75E-14, 1.03E-08, 6.78E-12), respectively. Higgins found a 3.226-fold decrease in mRNA expression of ATP6V1A in KIRC tissues. Yusenko, Gumz, and Jones observed significant down-expression in ATP6V1B1 mRNA in KIRC tissues. Down-regulation of mRNA expression of ATP6V1G3 was also found in KIRC tissues. Gumz also found that mRNA expression of ATP6V1H in KIRC was down-expression compared to normal tissues.

**Table 1:** Transcription expression of ATP6V1s family members between KIRC and normal kidney tissues (Oncomine).

	Types of KIRC VS. kidney	Fold Change	P-value	t-test	Ref
ATP6V1A					
	Kidney renal clear cell carcinoma	−2.403	4.75E−14	−13.994	Beroukhim Renal
	Kidney renal clear cell carcinoma	−3.226	0.0000126	−8.489	Higgins Renal
ATP6V1B1					
	Kidney renal clear cell carcinoma	−28.847	7.37E−10	−13.133	Yusenko Renal
	Kidney renal clear cell carcinoma	−28.998	7.51E−09	−12.854	Gumz Renal
	Kidney renal clear cell carcinoma	−13.706	1.03E−08	−14.916	Beroukhim Renal
	Kidney renal clear cell carcinoma	−5.428	7.32E−16	−16.622	Jones Renal
ATP6V1G3					
	Kidney renal clear cell carcinoma	−6.654	0.00000942	−8.388	Lenburg Renal
ATP6V1H					
	Kidney renal clear cell carcinoma	−2.276	6.78E−12	−13.296	Beroukhim Renal
	Kidney renal clear cell carcinoma	−2.009	1.95E−07	−7.814	Gumz Renal

**Figure 3:**
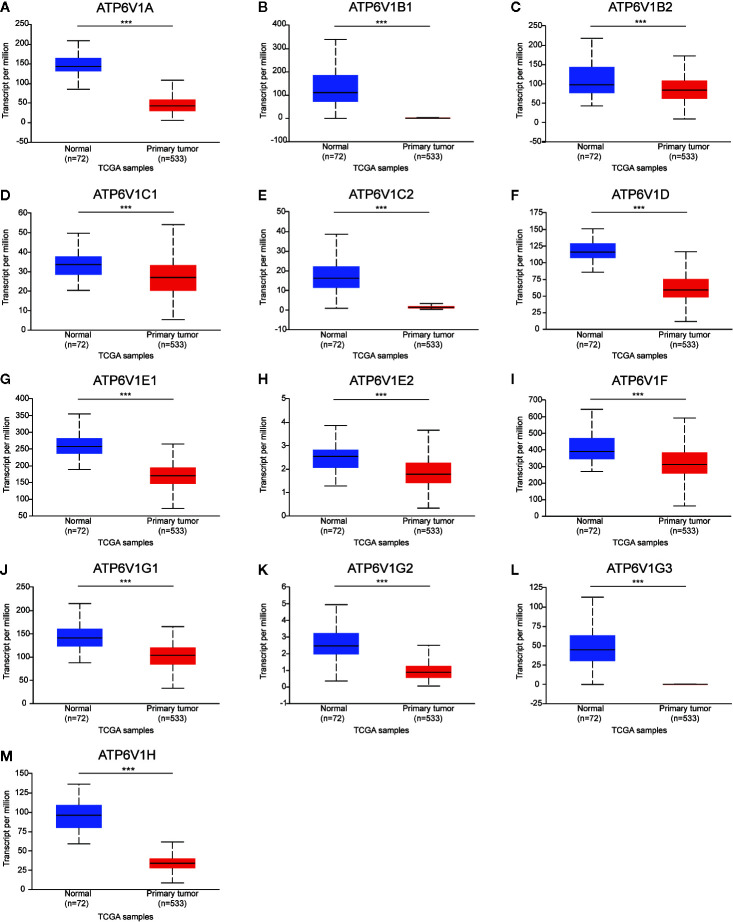
Low mRNA expressions of different ATP6V1s family members in patients with KIRC and normal kidney tissues (TCGA database). The mRNA expressions of different ATP6V1s family members were significantly down-regulated in patients with KIRC from the TCGA database **(A-M)**. ***p < 0.001.

Next, the mRNA expression patterns of ATP6V1s family members were further measured by the TCGA database. Consistent from the Oncomine database, as was shown in **Figure 3**, compared to normal samples mRNA expressions of all ATP6V1 members were significantly down-regulated in KIRC tissues.

### Protein Expression of Different ATP6V1s Family Members in Patients With KIRC

**Figure 4:**
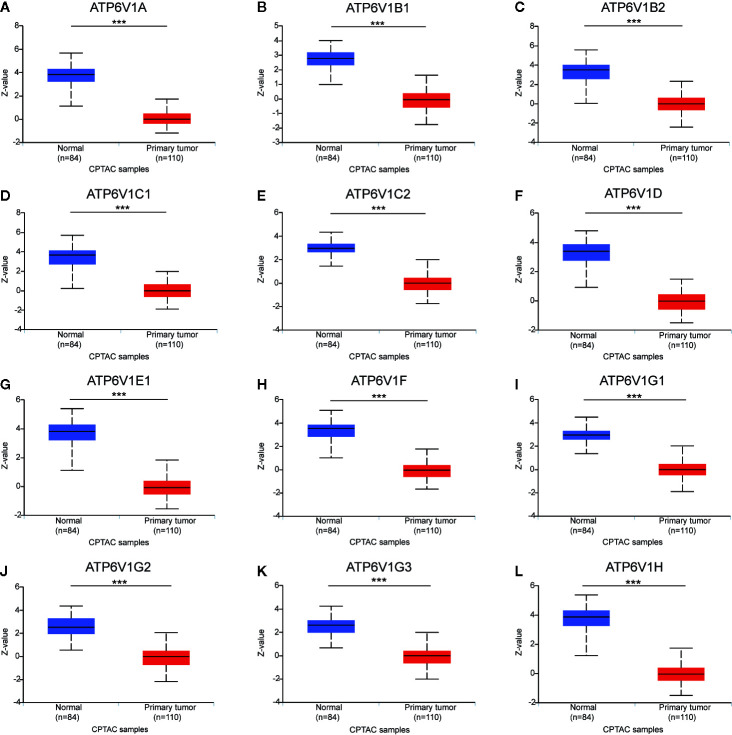
Protein expression of ATP6V1s family members in KIRC tissues and normal kidney tissues (CPTAC database). Protein expressions of different ATP6V1s family members were significantly down-regulated in patients with KIRC from the CPTAC database **(A–L)**. ***p < 0.001.

**Figure 5:**
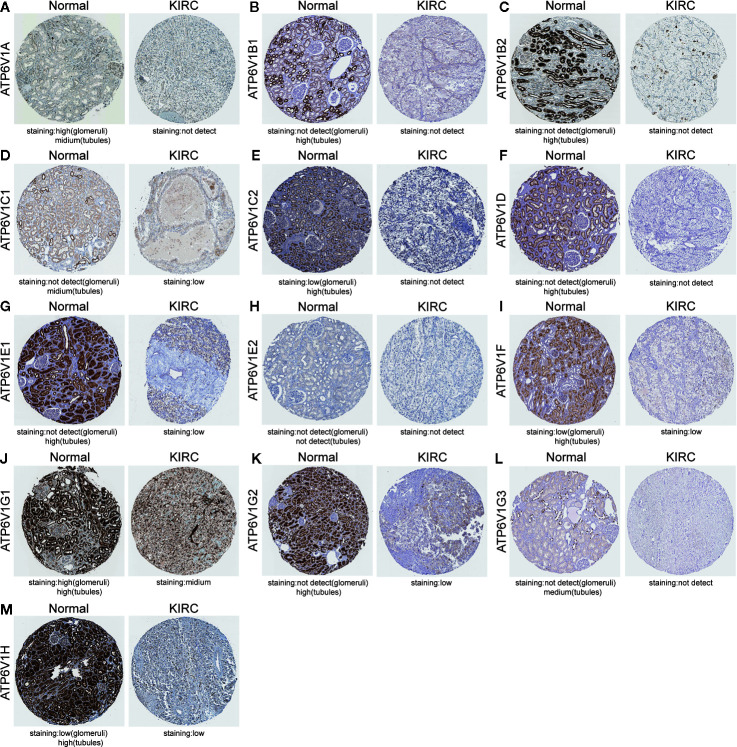
Representative immunohistochemistry images of different ATP6V1s family members in KIRC tissues and normal kidney tissues (HPS database). Except for ATP6V1E2, the protein expression of ATP6V1S family members were detected in normal kidney tissues, while the protein expressions were significantly down-regulated in KIRC tissues **(A–G**, **I–M)**. Protein expression of ATP6V1E2 was not detected both at normal and KIRC tissues **(H)**.

After analyzing the mRNA expression of ATP6V1s family members in KIRC, we explored the protein expression of ATP6V1s family members in KIRC by CPTAC and the Human Protein Atlas. As shown in **Figure 4**, Protein expression of all ATP6V1s family members was lower in KIRC tissues compared to the normal tissues by CPTAC. Similar results appeared by CPTAC analysis, ATP6V1s proteins were low expressions in KIRC tissues by HPA (**Figure 5**). Low protein expressions of ATP6V1A, ATP6V1B1, ATP6V1B2, ATP6V1C1, ATP6V1C2, ATP6V1D, ATP6V1E1, ATP6V1F, ATP6V1G1, ATP6V1G2, ATP6V1G3, and ATP6V1H were found in KIRC tissues, while their medium and high protein expressions were observed in normal kidney tissues. Negative protein expression of ATP6V1E2 was observed both at normal kidney tissues and KIRC tissues (**Figure 5**). Taken together, our results showed that protein expressions of ATP6V1s family members were significantly low-expressed in patients with KIRC.

Generally, all the results above showed that ATP6V1s were under-expressed in KIRC both in the transcriptional and protein expressions.

### Function Enrichment of ATP6V1s Family Members in KIRC

**Figure 6:**
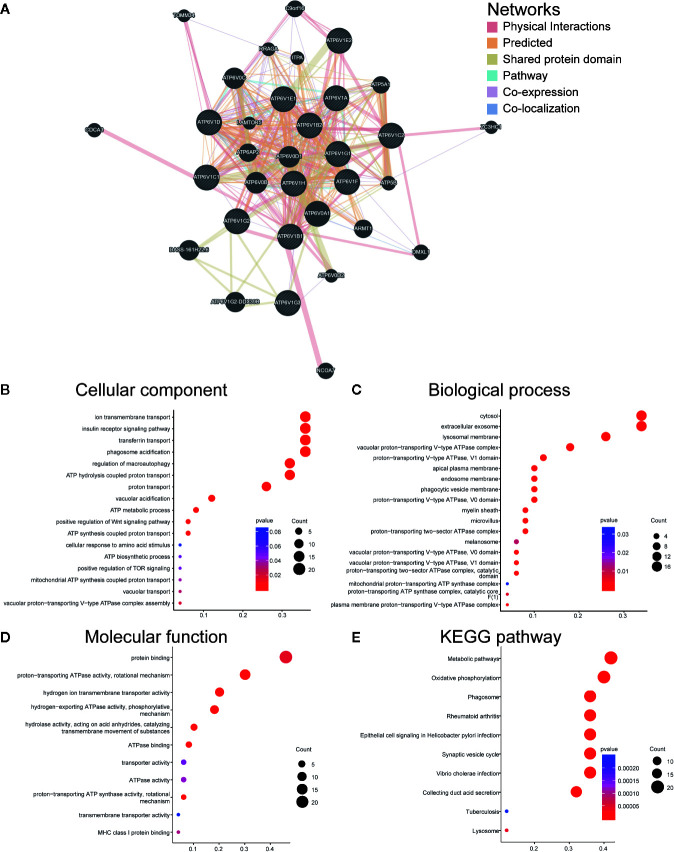
Function enrichment of ATP6V1s family members in KIRC. **(A)** Network of ATP6V1s family members and their 20 related genes was analyzed by GeneMANIA. **(B)** Cellular component; **(C)** Biological processes; **(D)** Molecular functions; **(E)** KEGG pathway analysis.

We constructed a network of ATP6V1s family members and their 20 related genes by GeneMANIA (**Figure 6A**). Proteins that interact with ATP6V1s family members include ATP6V0A1, ATP6V0B, ATP6V0 ATP6V0D1, ATP6V0C, ATP6V1G2-DDX39B, DASS-161H22.6, ARMT1, ATP6AP2, ATP5A1, and C9orf16.

GO functions and pathways of ATP6V1s and their 20 related genes were analyzed by DAVID. The biological processes such as GO: 0090383 (phagosome acidification), GO: 0033572 (transferrin transport), GO: 0015991 (ATP hydrolysis coupled proton transport), GO: 0008286 (insulin receptor signaling pathway), and GO: 0016241 (regulation of macroautophagy) were remarkably regulated by the ATP6V1s in KIRC (**Figure 6B**). Cellular components, including GO: 0016471 (vacuolar proton-transporting V-type ATPase complex), GO: 0005765 (lysosomal membrane), GO: 0033180 (proton-transporting V-type ATPase, V1 domain), GO: 0033179 (proton-transporting V-type ATPase, V0 domain), and GO: 0016469 (proton-transporting two-sector ATPase complex) were significantly associated with the ATP6V1s alterations (**Figure 6C**). Besides, ATP6V1s also prominently affected the molecular functions (**Figure 6D**), such as GO: 0046961 (proton-transporting ATPase activity, rotational mechanism), GO: 0008553 (hydrogen-exporting ATPase activity, phosphorylative mechanism), GO: 0015078 (hydrogen ion transmembrane transporter activity), GO: 0016820 (hydrolase activity, acting on acid anhydrides, catalyzing transmembrane movement of substances) and GO: 0051117 (ATPase binding).

In KEGG analysis, these pathways including hsa04966 (Collecting duct acid secretion), hsa05110 (Vibrio cholerae infection), hsa04721 (Synaptic vesicle cycle), hsa05120 (Epithelial cell signaling in Helicobacter pylori infection), and hsa00190 (Oxidative phosphorylation) were associated with the functions of ATP6V1s in KIRC (**Figure 6E**).

### Association of mRNA Expression of ATP6V1s Family Members With Immune Infiltration Level in KIRC

**Figure 7:**
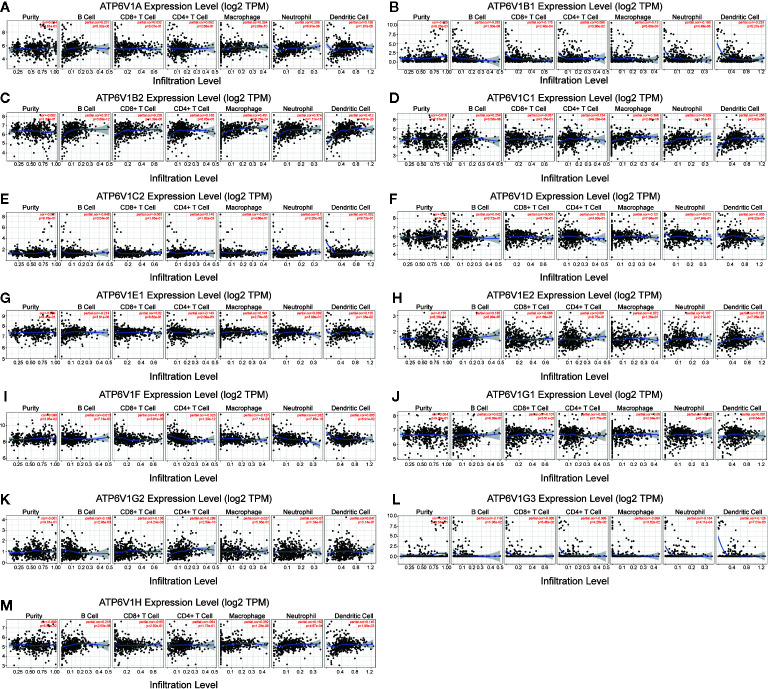
Association of mRNA expression of ATP6V1s family members with immune infiltration level in KIRC. The mRNA expression of ATP6V1s family members was significantly related to the immune infiltration level in KIRC **(A–M)**.

Then, we investigated whether mRNA expression of ATP6V1s family was correlated with immune infiltration levels in KIRC from the TIMER database. The results showed that the mRNA expressions of ATP6V1D, ATP6V1F, and ATP6V1F were obviously related to tumor purity (**Figures 7F, H, I**). The correlation of mRNA expression of ATP6V1A, ATP6V1B1, ATP6V1B2, ATP6V1C1, ATP6V1E1, ATP6V1E2, ATP6V1G2, ATP6V1G3, and ATP6V1H with B cell was statistically significant (**Figures 7A–D, G, H, K–M**). While mRNA expression of ATP6V1B1, ATP6V1B2, ATP6V1F, ATP6V1G1, and ATP6V1G2 was obviously related to CD8^+^ T cell (**Figures 7B, C, I–K**). In addition, mRNA expression of ATP6V1B1, ATP6V1B2, ATP6V1C1, ATP6V1C2, ATP6V1E1, ATP6V1F, ATP6V1G2, and ATP6V1G3 had significant correlations with infiltrating levels of CD4^+^ T cells in KIRC (**Figures 7B–F, I, K, L**). The mRNA expressions of ATP6V1A, ATP6V1B1, ATP6V1B2, ATP6V1C1, ATP6V1D, ATP6V1E1, ATP6V1F, ATP6V1G3, and ATP6V1H were obviously related to macrophage (**Figures 7A–D, F, G, I, L, M**). The mRNA expressions of ATP6V1A, ATP6V1B1, ATP6V1B2, ATP6V1C1, ATP6V1C2, ATP6V1E2, ATP6V1F, ATP6V1G3, and ATP6V1H were obviously related to neutrophil infiltration (**Figures 7A-E, H, I, L, M**). The mRNA expressions of ATP6V1A, ATP6V1B1, ATP6V1B2, ATP6V1C1, ATP6V1E2, ATP6V1G3, and ATP6V1H were obviously related to dendritic cell infiltration (**Figures 7A–D, H, L, M**).

### Association of mRNA and Protein Expression of ATP6V1s Family Members With Clinicopathological Features of KIRC Patients

**Figure 8:**
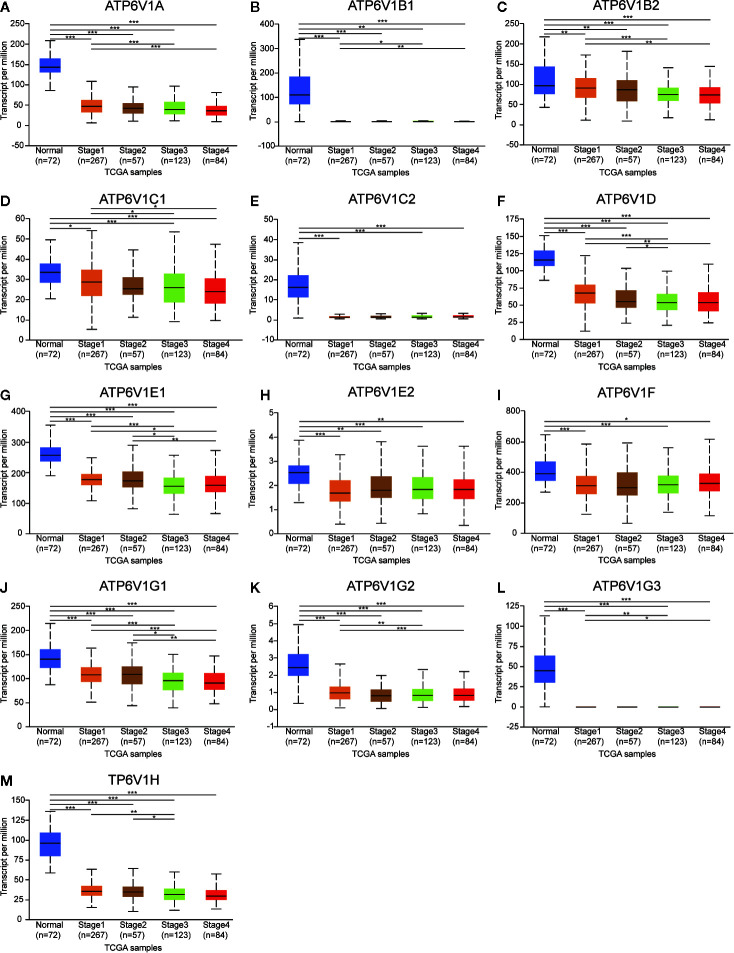
Association of mRNA expression of ATP6V1s family members with cancer stages of KIRC patients. Boxplot showing the mRNA expression of ATP6V1s family members in normal individuals or in KIRC patients in stages 1, 2, 3 or 4 **(A–M)**. *p < 0.05; **p < 0.01; ***p < 0.001.

Next, the relationship between mRNA expression of ATP6V1s family members with clinicopathological parameters of KIRC patients was analyzed by CTGA, including individual cancer stages and nodal metastasis status. As was shown in **Figure 8**, mRNA expressions of ATP6V1s family members were remarkably correlated with cancer stages, and patients who were in more advanced cancer stages tended to express lower mRNA expression of ATP6V1s. Compared to normal tissues, the mRNA expression of ATP6V1s family members was significantly lower in stage 1, stage 2, stage 3, and stage 4. While there was no significant difference in mRNA expression between stage 2 and normal tissues. That may be due to the small sample size in stage 2 (only 87 samples).

**Figure 9:**
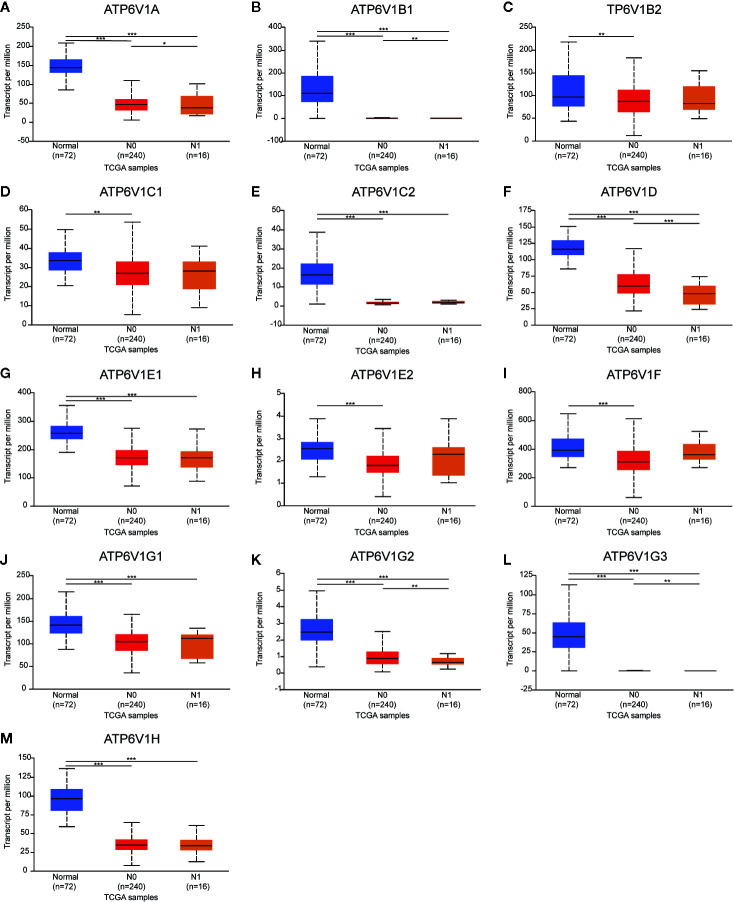
Association of mRNA expression of ATP6V1s family members with nodal metastasis status of KIRC patients. Boxplot showing the mRNA expression of ATP6V1s family members in normal individuals or in KIRC patients in nodal metastasis status N0 or N1 **(A–M)**. *p < 0.05; **p < 0.01; ***p < 0.001.

Then, we analyzed the relationship mRNA expression of ATP6V1s family members with nodal metastasis status of KIRC patients. As shown in **Figure 9**, mRNA expressions of ATP6V1A, ATP6V1B1, ATP6V1D, ATP6V1G2, and ATP6V1G3 were significantly related to nodal metastasis status (**Figures 9A, B, F, K, L**). However, the relationship between mRNA expressions of ATP6V1B2, ATP6V1C1, ATP6V1C2, ATP6V1E1, ATP6V1E2, ATP6V1F, ATP6V1G1, and ATP6V1H with nodal metastasis status without a significant statistic difference (**Figures 9C–E, G–J, M**).

**Figure 10:**
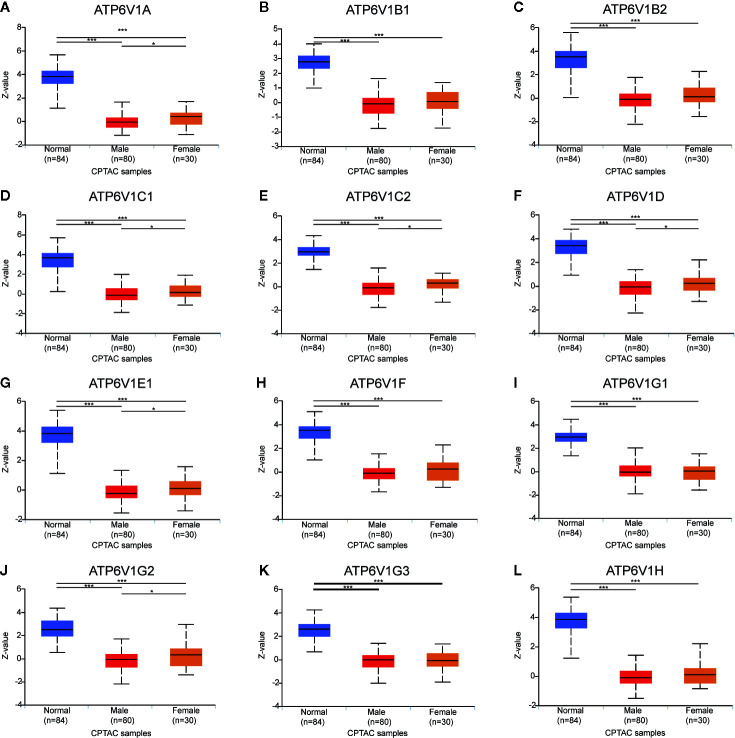
Association of protein expression of ATP6V1s family members with the gender of KIRC patients. Boxplot showing relative expression of ATP6V1s family members in normal individuals of either gender and male or female LIHC patients, respectively **(A–L)**. *p < 0.05; ***p < 0.001.

The relationship between protein expressions of ATP6V1s family members with the gender of KIRC patients was analyzed by CPTAC. The protein expressions of ATP6V1A, ATP6V1C1, ATP6V1C2, ATP6V1D, ATP6V1E1, and ATP6V1G2 in female were significantly higher than the male with statistic difference, while the difference between ATP6V1B1, ATP6V1B2, ATP6V1F, ATP6V1G1, ATP6V1G3, and ATP6V1H was not remarkable (**Figure 10**).

In brief, mRNA expression of part TP6V1s members was associated with clinicopathological parameters of KIRC patients.

### Prognostic Value of mRNA Expression of ATP6V1s Family Members in KIRC Patients

**Figure 11:**
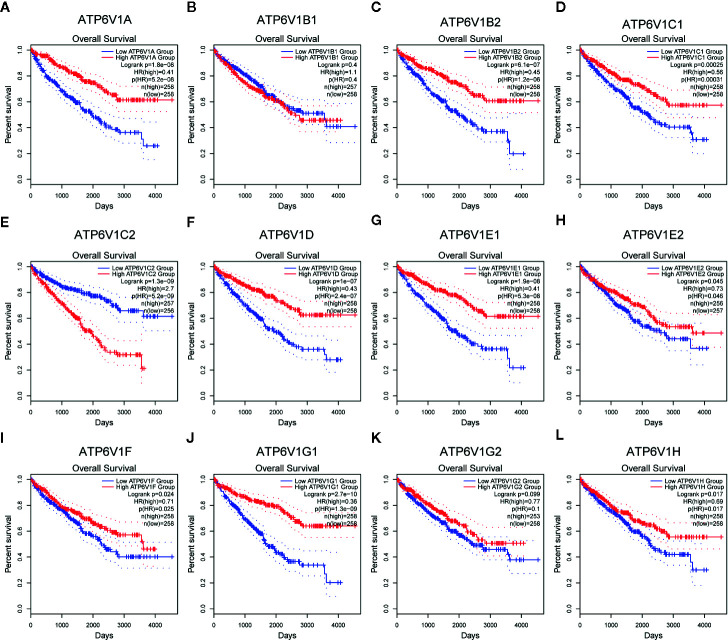
Prognostic value of mRNA expression of ATP6V1s family members in KIRC patients. Survival curves comparing the high and low expression of ATP6V1s family members in KIRC patients in GEPIA **(A–L)**.

The association between mRNA expression of ATP6V1s family members and prognosis of KIRC patients was analyzed by Kaplan–Meier Plotter. As were shown in **Figures 11A, C–J, L**, lower mRNA expression of ATP6V1A (HR(high)=0.41, and Log-rank p=1.8e-08), ATP6V1B2 (HR(high)=0.45, and Log-rank p=6.1e-07), ATP6V1C1 (HR(high)=0.56, and Log-rank p=2.5e-05), ATP6V1C2 (HR(high)=2.7, and Log-rank p=1.3e-09), ATP6V1D (HR(high)=0.43, and Log-rank p=1e-07), ATP6V1E1 (HR(high)=0.41, and Log-rank p=1.9e-08), ATP6V1E2 (HR(high)=0.73, and Log-rank p=0.045), ATP6V1F (HR(high)=0.71, and Log-rank p=0.024), ATP6V1G1 (HR(high)=0.36, and Log-rank p=2.7e-10), ATP6V1H (HR(high)=0.69, and Log-rank p=0.017) were significantly associated with shorter OS of KIRC patients. However, mRNA expression of ATP6V1B1 and ATP6V1G2 showed no correlation with the prognosis of KIRC patients (**Figures 11B, K**). These results indicated mRNA expressions of part of ATP6V1s family members were significantly associated with the prognosis of KIRC patients, and they may be useful biomarkers for prediction of KIRC patients’ survival.

## Discussion

Many cytokines, hormones, and proteins are involved in the development and progression of KIRC. ATP6V1s as components of V-ATPase has been shown to participate in the development of multiple tumors, including KIRC. Even so, the role of ATP6V1s family members in the prognosis value of KIRC is still unclear. In this study, we analyzed the expression and prognostic value of different ATP6V1s family members in KIRC.

Results from our study showed that mRNA expressions of ATP6V1A, ATP6V1B1, ATP6V1D, ATP6V1F, ATP6V1G3, and ATP6V1H were significantly lower in KIRC tissues compared to normal tissues from the Oncomine database. However, mRNA expressions of all ATP6V1s family members were significantly down-regulated in KIRC tissues from the TCGA database. Besides, through analyzing the protein expression of ATP6V1s family members in KIRC by HPA and CPTAC, we found that protein expressions of ATP6V1A, ATP6V1B1, ATP6V1B2, ATP6V1C1, ATP6V1C2, ATP6V1D, ATP6V1E1, ATP6V1F, ATP6V1G1, ATP6V1G2, ATP6V1G3, and ATP6V1H were lower than normal tissues, and similar results were found by CPTAC. Next, it was found that proteins including ATP6V0A1, ATP6V0B, ATP6V0 ATP6V0D1, ATP6V0C, ATP6V1G2-DDX39B, DASS-161H22.6, ARMT1, ATP6AP2, ATP5A1, and C9orf16 interacted with ATP6V1s family members through PPI network analysis by GeneMANIA. The function enrichment of these genes was the collecting duct acid secretion. Subsequently, the association of mRNA and protein expression of ATP6V1s family members with clinicopathological parameters of KIRC patients was analyzed. The mRNA expressions of ATP6V1A, ATP6V1B1, ATP6V1B2, ATP6V1C1, ATP6V1D, ATP6V1E1, ATP6V1G1, ATP6V1G2, ATP6V1G3, and ATP6V1H were remarkably correlated with cancer stages, while mRNA expressions of ATP6V1A, ATP6V1B1, ATP6V1D, ATP6V1G2, and ATP6V1G3 were significantly related to nodal metastasis status. According to the results of data analysis in CPTAC, the protein expressions of ATP6V1A, ATP6V1C1, ATP6V1C2, ATP6V1D, ATP6V1E1, and ATP6V1G2 related to the gender of patients. Finally, analysis of the prognostic value of mRNA expression levels of ATP6V1s family members in KIRC patients revealed that lower mRNA expression of ATP6V1A, ATP6V1B2, ATP6V1C1, ATP6V1C2, ATP6V1D, ATP6V1E1, ATP6V1E2, ATP6V1F, ATP6V1G1, and ATP6V1H have shorter OS.

Abnormal ATPase subunit expression and dysregulated ATPase activity are closely related to the occurrence, proliferation, and invasion of various tumors (11, 12, 24–26). Numerous studies have shown that ATP6V1s family members are abnormally expressed in tumor tissues or tumor cell lines. Over-expression of ATP6V1C1 had been found in oral squamous cell carcinoma (27–29). In human pancreatic cancer, V-ATPase was significantly overexpressed (30). The expression of ATP6V1A in gastric cancer tissue is significantly higher than that in normal tissue, and its expression is related to histological grade, lymph node metastasis, and vascular invasion. Knocking down the expression of ATP6V1A *in vitro* inhibits the proliferation and invasion ability of gastric cancer cells (31). ATPase promotes the formation of a slightly alkaline microenvironment around tumor cells, which is beneficial to tumor cell proliferation (32). ATP6V1C1 promotes the growth of breast cancer by activating the mTORC1 pathway and promotes bone metastasis by activating V-ATPase (33). ATP6V1C1 may promote breast cancer growth and bone metastasis by regulating lysosomal V-ATPase activity *in vivo* and *in vitro* (34). Down-regulate the expression of ATP6V0C and ATP6V1A, which inhibits the activity of V-ATPase, reduces the invasiveness of liver cancer cells (35).

Studies have shown that ATP6V1C1 could be used as a marker of diagnosis and prognosis in oral squamous cell carcinoma (29). In glioblastoma, high expression of ATP6V1G1 is associated with poor prognosis (36).

Obviously, there were some limitations to this study. First, all the data analyzed was based on the online databases in silicon, further *in vivo* and *in vitro* studies are required to verify these findings. Second, the underlying mechanisms of distinct ATP6V1s in KIRC is still unknown. Further experiments are worth to reveal the detailed mechanism between ATP6V1s and KIRC. Besides, this study was only a retrospective study, further detailed prospective results will support each other.

In conclusion, our results showed that underexpression of ATP6V1s members in KIRC was found on distinct public databases. Moreover, ATP6V1s were significantly associated with individual cancer stage, nodal metastasis status, and patient’s gender. Furthermore, high expressions of ATP6V1s were significantly related with longer OS in KIRC patients. All in a word, ATP6V1s family members could be a potential target in the development of anti-KIRC therapeutics and an efficient marker of the prognostic value of KIRC.

## Data Availability Statement

The datasets presented in this study can be found in online repositories. The names of the repository/repositories and accession number(s) can be found in the article/supplementary material.

## Author Contributions

XL and HL analyzed the data. CY and LL suggested online tools. SD and ML designed the project, selected the analyzed results, and wrote the paper. All authors contributed to the article and approved the submitted version.

## Funding

The present study was supported by the National Natural Science Foundation of China (Grant No. 81200521).

## Conflict of Interest

The authors declare that the research was conducted in the absence of any commercial or financial relationships that could be construed as a potential conflict of interest.
